# Identifying the early 2000s hiatus associated with internal climate variability

**DOI:** 10.1038/s41598-018-31862-z

**Published:** 2018-09-11

**Authors:** Xin-Gang Dai, Ping Wang

**Affiliations:** 1 RCE-TEA, Institute of Atmospheric Physics, Chinese Academy of Sciences, Beijing, 100029 China; 2 Institute of Atmospheric Composition, Chinese Academy of Meteorological Sciences, Beijing, 100081 China

## Abstract

This study focuses on re-examining the early 2000s hiatus and the associated key components of the global mean surface temperature (GMST) using multiscale statistics for five well-known gridded surface temperature and two reanalysis datasets. The hiatus is characterized as a near-zero trend on the decadal scale corresponding to the maximum P-value via an F-test in statistics. The results reveal that the hiatus exists in both the GMST and global mean air temperature (GMAT) time series, rather than in global warming component, which has maintained an approximately constant rate of change of approximately 0.08 °C/decade over the past three decades. The hiatus’s duration is different from that of time series such as 2002–2012/2001–2013/2002–2014 in HadCRUT4, NOAA-old, ERA-Interim and NCEP-R2. The newly gridded datasets with data infilling or bias correction for interpreting the sea surface temperature (SST) measurement from the old versions show a slightly higher trend from 2002–2012 than the hiatus, which is thus regarded as a slowdown. Comparison suggests that the hiatus should be during the period 2002–2012. Orthogonal wavelet decomposition of the temperature time series shows that the hiatus was merely a decadal balance between cooling from interannual variability and global warming, in addition to weak warming from interdecadal and multidecadal climate oscillations. In addition, the evolutions of the GMST’s interannual composites are well coincided with Niño3.4 SST anomalies, which is consistent with the numerical simulation performed by Kosaka and Xie in 2013. Hence, it is the anomalous El Niño Southern Oscillation (ENSO) events in the early 2000s that caused the hiatus despite a constant rate of global warming and the maximum magnitude of the multidecadal composite that led to the limited contribution to the trend during this period. The multidecadal composite follows a downward path, which implies that future climate conditions will likely rely on competition between multidecadal cooling and global warming if the multidecadal climate cycle repeats, as was experienced during the second half of the twentieth century.

## Introduction

Global warming has been attributed to persistent increase in atmospheric greenhouse gasses (GHGs), especially in CO_2_, since the beginning of the Industrial Revolution^1,2^. Nevertheless, the upward trend in the global mean surface temperature (GMST) slowed or even paused during the first decade of the twenty-first century^3^, even though CO_2_ levels continued to rise and reached nearly 400 ppm in 2013 (https://www.climate.gov/news-features/understanding-climate/2013-state-climate-carbon-dioxide-tops-400-ppm). This episode has typically been termed the global warming hiatus (GWH)^4^. The GWH is often attributed to internal climate variability, external forcing, or both. Recent cooling in the middle and eastern regions of the tropical Pacific has seemingly involved a phase change of the Interdecadal Pacific Oscillation (IPO)^5,6^ accompanying intensified trade winds^7,8^. The GWH may also be associated with an increase in aerosols in the stratosphere during the period 2000–2010 because aerosols can increase optical depth, which generates countervailing forces against global warming^9,10^. The GWH may also be explained in part by extensive heat uptake by the deep ocean^11,12^ or an extremely low number of sunspots during the latest solar activity cycle^13,14^.

Since the surface of the globe warms unevenly, the GMST is sensitive to data coverage, interpolation, observation bias, and even the techniques used to interpret sea surface temperature (SST) measurements^15,16^ because the GMST anomaly is the offset of positive and negative anomalies at every grid on the surface, and any spurious increase in the temperature resulting from insufficient data coverage or the interpretation technique in SST observation that could influence the GMST. Two noticeably updated datasets were released near the end of the hiatus. The first dataset (hereafter Cowtan & Way)^17^ was updated via infilling over the Arctic region and the African continent from the Hadley Centre–Climatic Research Unit Version 4 (HadCRUT4)^17–20^, and the second, which was a new bias-corrected dataset (hereafter NOAA-new)^21^, was updated from an older with infilling and bias-correction from the National Oceanic and Atmospheric Administration (NOAA) global surface temperature dataset (hereafter NOAA-old)^22,23^. The presence of the hiatus in HadCRUT4 was regarded as the result of sparse data over the Arctic region and African continent, which suppressed the GMST trend^17^, while the hiatus was considered to even possibly be an artifact created by data biases in the NOAA-old dataset or its earlier version^21^. These problems affect observation and the definition and identification of the hiatus^24,25^. Thus far, two approaches have been used to identify the hiatus or slowdown^26^: (i) when the trend in GMST is observed to be approximately zero or nonsignificant at the 0.05 significance level and (ii) when the decadal trend is observed to be less than the long-term trend, in addition to the identification of the change point (CP) in the trend^27^. The first approach is a well-known general measure for trend in scientific literature. The second approach seems to be unsuitable for assessing the hiatus or slowdown at a decadal scale because the decadal trend during the hiatus period is compared with the long-term trend from 1951 to 2012^1^, which can be influenced by interdecadal and multidecadal climate oscillations. The additional approach involves identifying the CP in the trend and may be difficult to use in identifying the early 2000s hiatus because the formation of the hiatus (in terms of trend) occurred gradually from the previous maximum warming trend to the following minimum trend. Hence, arguments seem to result not only from the biases in the SST measurements^16,21^ or sparse data in the datasets^17,20^ but also from the absence of properly quantitative assessments of the contributions of multiscale components to GMST, which are easily tied to the mechanisms responsible for the hiatus (e.g., internal factors or external forcing). This study first quantitatively re-examines the existence of the hiatus in the HadCRUT4, Cowtan & Way, NOAA-old, NOAA-new, NASA GISS Surface Temperature (GISTEMP)^23^, ERA-Interim^28^ and NCEP-DOE Reanalysis 2 (hereafter NCEP-R2)^29^ series and the reanalysis global mean air temperature (GMAT) series. Furthermore, it compares the potential hiatus in the gridded instrumental data and reanalysis datasets to obtain an overarching statement, where the former series are based on some kind of three dimensional interpolation methods, and the latter are produced by a four-dimensional data assimilation system with many more four-dimensional observations (regular and irregular) than the former, leading to datasets that are dynamically consistent. Second, the contributions from the multiscale components are analyzed using orthogonal wavelet decomposition^30^ to distinguish the key components in terms of their contributions to the hiatus and their potential links with the El Niño/Southern Oscillation (ENSO) cycle in the equatorial eastern Pacific^31^.

## Results

**Figure 1:**
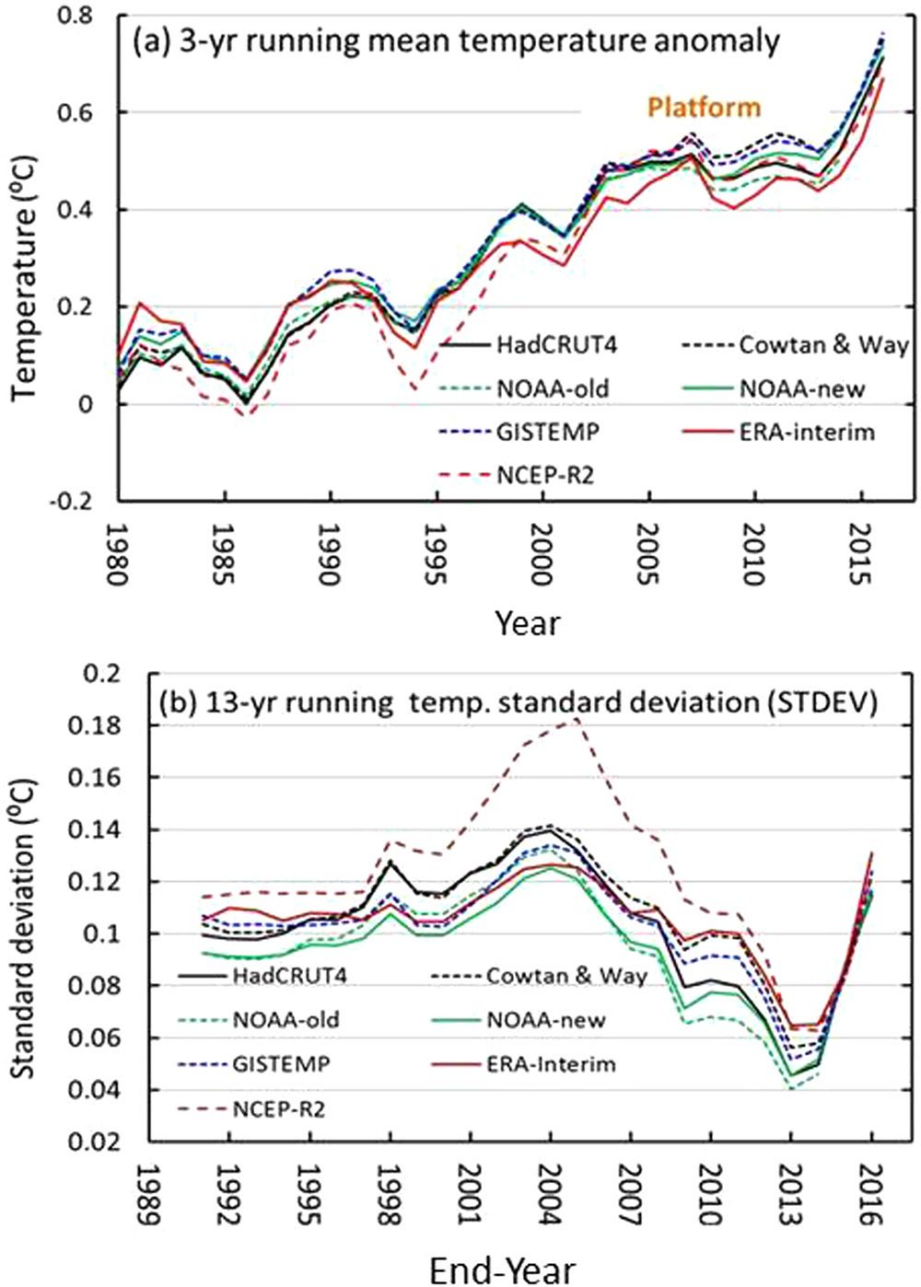
Global mean surface temperature (GMST) anomalies with reference to the period 1961–1990 and their running standard deviations (STDEVs). (**a**) The 3-yr running mean GMSTs from 1980–2016 and (**b**) the 13-yr running STDEVs in GMSTs from the HadCRUT4, Cowtan & Way, NOAA-old, NOAA-new, GISTEMP, ERA-Interim and NCEP-R2 datasets. The figures were generated using Excel (https://www.microsoft.com/zh-cn/download/office.aspx).

The hiatus or slowdown can be identified by comparing the statistical characteristics of the GMST/GMAT series during the early 2000s with those for the decades during the late twentieth century (e.g., decadal trends and standard deviations). Figure 1a shows that a decadal platform appears in all of the 3-yr (year) smoothed GMST series of the HadCRUT4, Cowtan & Way, NOAA-old, NOAA-new, GISTEMP, ERA-Interim and NCEP-R2 datasets since the 21^st^ century after the rapid warming period in the two or three decades of the late twentieth century. Differences among these series can be found throughout these platforms, in which the GISTEMP contains the maximum values and the ERA-Interim contains the minimum values. The NOAA-new dataset has values greater than the NOAA-old dataset, while the Cowtan & Way dataset has values greater than the HadCRUT4 dataset, which indicates that infilling and bias correction in the datasets increase the temperature, especially during the early 2000s, probably due to rapid warming in the Arctic region^32,33^. In addition, the decadal platform corresponds to a minimum standard deviation (STDEV) from 2001–2013, where the datasets of NOAA-new and Cowtan & Way are greater than those of the NOAA-old and HadCRUT4 (Fig. 1b), which reflects the effects of infilling or bias correction on the STDEV. In addition, the NCEP-R2 shows the largest STDEV of all the datasets, especially circa 2004. Further calculations show that the STDEVs from 2002–2012 and 2000–2014 are all larger than those from 2001–2013. Thus, the platform represents a unique period in which the interannual variabilities of the GMSTs become the weakest throughout all the seven series since the 1980s.

**Figure 2:**
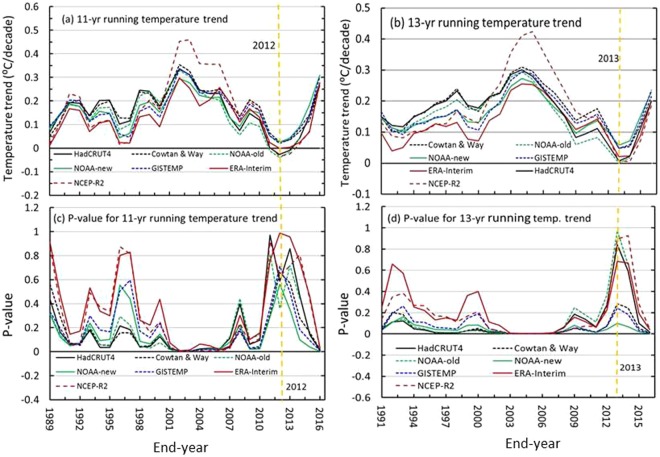
Running global mean surface temperature (GMST) trends with P-values obtained via an F-test. (**a**) 11-yr running trend; (**b**) 13-yr running trend; (**c**) P-values for the 11-yr running trends; and (**d**) P-values for the 13-yr running trends. The GMSTs refer to the HadCRUT4, Cowtan & Way, NOAA-old, NOAA-new, GISTEMP, ERA-Interim and NCEP-R2 datasets, in sequence. The figures were generated using Excel (https://www.microsoft.com/zh-cn/download/office.aspx).

However, whether the early 2000s temperature platform can be regarded as the early 2000s hiatus or slowdown requires further assessment of the temperature trends surrounding this period at different scales. Linear trends in the seven series are estimated by using moving windows with widths of 11, 12, 13, 14, and 15 years using linear regressions based on the ordinary least squares (OLS) method (see data and method) to determine the location and duration of the hiatus. Figure 2a shows that the 11-yr trends in the series all reached their minimums during the period 2002–2012, and the minimum trends in the ERA-Interim are near zero, while those of NOAA-new, Cowtan & Way and GISTEMP are slightly greater than zero, corresponding to the maximum P-values obtained via an F-test and representing the most nonsignificant trends over the period. Those from the HadCRUT4, NOAA-old and NCEP-R2 are negative (below bottom axis; Fig. 2a), which correspond to a valley between two P-value peaks (Fig. 2c), implying that the period (2002–2012) for the minimum trends is shorter than the period that should be expected in the three series, except in the ERA-Interim series, in which it has the smallest trend (−0.0011 °C/decade) and the largest P-value (0.9885) among the series (Fig. 2c). For the 13-yr window, the trends of HadCRUT4, NOAA-old and NCEP-R2 become 0.0076 °C/decade, 0.0010 °C/decade and 0.0047/decade, with P-values of 0.8393, 0.9758 and 0.9271, respectively, indicating that the trends are all most nonsignificant (Fig. 2b,d). Hence, the minimum trends of ERA-Interim, HadCRUT4, NOAA-old and NCEP-R2 may become the potential candidates for the early 2000s hiatus, while those of NOAA-new, Cowtan & Way and GISTEMP can to some extent be regarded as a slowdown. However, further tests at different scales under moving windows are needed to identify whether the minimum trends in the windows of 11 years and 13 years are the smallest relative to longer or shorter windows.

**Figure 3:**
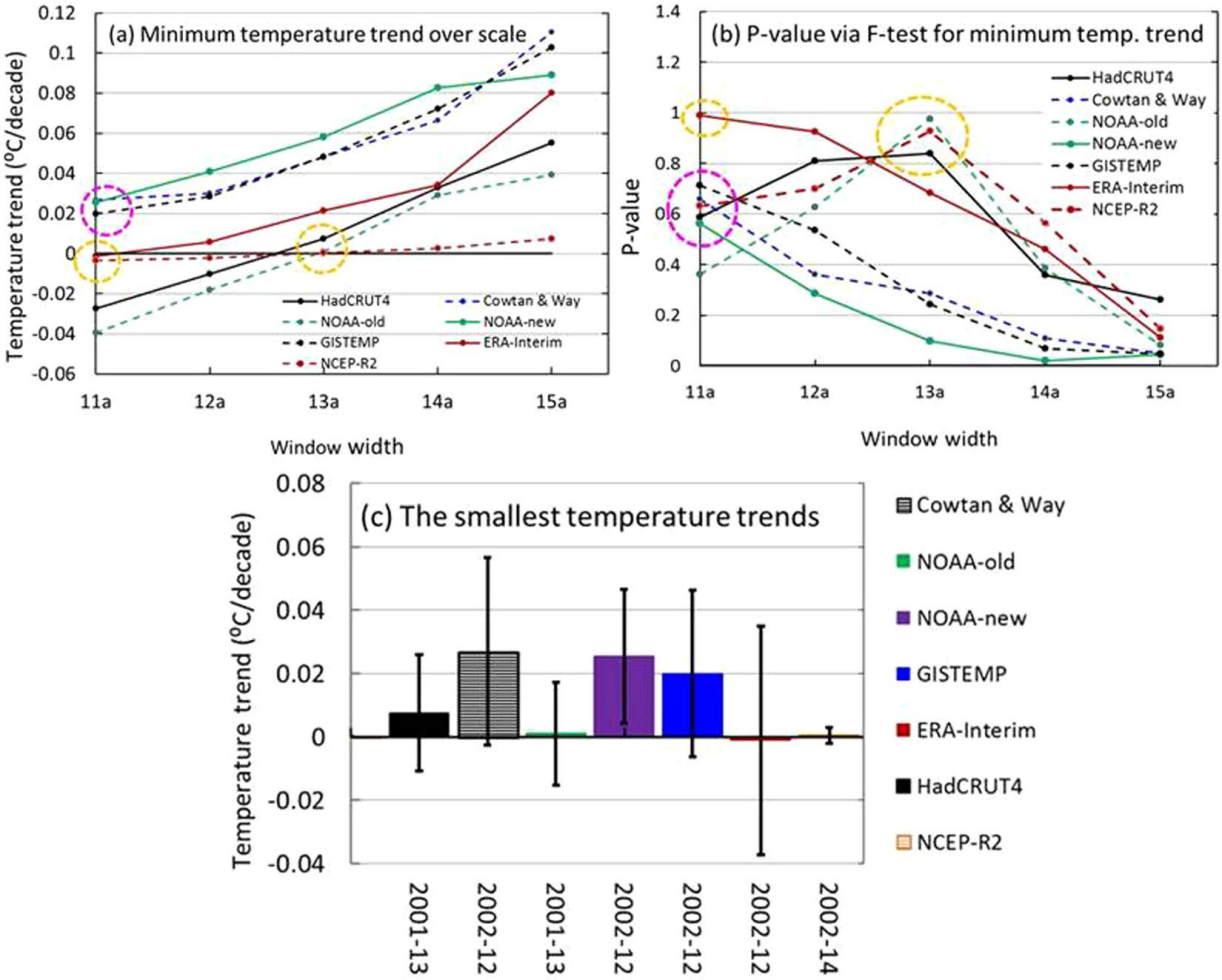
The minimum trends in global mean surface temperature (GMST) with five window widths for the HadCRUT4, Cowtan & Way, NOAA-old, NOAA-new, GISTEMP, ERA-Interim and NCEP-R2 datasets. (**a**) The minimum trends in GMST with the 11-yr, 12-yr, 13-yr, 14-yr, and 15-yr windows; (**b**) the corresponding P-values obtained via an F-test; and (**c**) the smallest trends that correspond to the points circled by dashed yellow and pink rings in (**a**) and (**b**), as well as their uncertainties. The figures were generated using Excel (https://www.microsoft.com/zh-cn/download/office.aspx).

Figure 3a shows that the minimum trends within a window increase with the width of the window, which corresponds to a decreasing in the P-value for the NOAA-new, GISTEMP, Cowtan & Way and ERA-Interim datasets; however, the P-values for the HadCRUT4, NOAA-old and NCEP-R2 first increase to their maximums in the 13-yr window (2001–2013; 2002–2014 for NCEP-R2) and then decrease (Fig. 3b). The weakest trend (of near zero) for the period 2002–2012 comes from the ERA-Interim, with a P-value much greater than those of the other datasets, while HadCRUT4 and NOAA-old have the weakest trends for the period 2001–2013, and the NCEP-R2 dataset from 2002–2014 had the largest P-value over the window. Hence, the four smallest trends, circled in yellow, from 2002–2012/2001–2013/2002–2014 should be regarded as the hiatus, while the others, circled in pink, from 2002–2012 (Fig. 3a) may be regarded as slowdowns, with maximum P-values less than those of the hiatus (Fig. 3b). Figure 3c depicts the minimum trends in yellow and pink circles in Fig. 3a along with uncertainties.

The smallest trends and durations and their uncertainties are listed in Table 1. By comparing the trends with their uncertainties, one can see that the trends with the P-values can be separated into two groups: 1) those for HadCRUT4, NOAA-old, ERA-Interim and NCEP-R2, with trend norms below 0.01 °C/decade and P-values above 0.8, and 2) those for NOAA-new, GISTEMP and Cowtan & Way, with trend norms above 0.01 °C/decade and P-values below 0.8. Hence, a group’s trends over the period 2002–2012 or 2001–2013 or 2002–2014 (circled in yellow in Fig. 3a) should be regarded as the early 2000s hiatuses, and the rest (circled in pink in Fig. 3a) over the period 2002–2012 may be referred to as slowdowns. The hiatus periods we found differ from those estimated by Easterling and Wehner due to the limitations of the temperature series length they used^34^.

**Table 1:** The smallest trends in the global mean surface/air temperature (GMST/GMAT) time series over a period (duration) with uncertainty, P-value and duration.

Time series	Temperature	Trend °C/decade	Uncertainty °C/decade	P-value	Duration
HadCRUT4	GMST	0.0076	± 0.0183	0.8393	2001–2013
Cowtan & Way	GMST	0.0482	± 0.0296	0.6602	2002–2012
NOAA-old	GMST	0.0010	± 0.0212	0.9758	2001–2013
NOAA-new	GMST	0.0255	± 0.0212	0.5626	2002–2012
GISTEMP	GMST	0.0200	± 0.0264	0.7137	2002–2012
ERA-Interim	GMST	−0.0011	± 0.0361	0.9885	2002–2012
NCEP-R2	GMST	0.0179	± 0.0025	0.9271	2002–2014
ERA-Interim	GMAT	−0.0001	± 0.0361	0.9885	2002–2012
NCEP-R2	GMAT	0.0082	± 0.0337	0.9057	2002–2012

**Figure 4:**
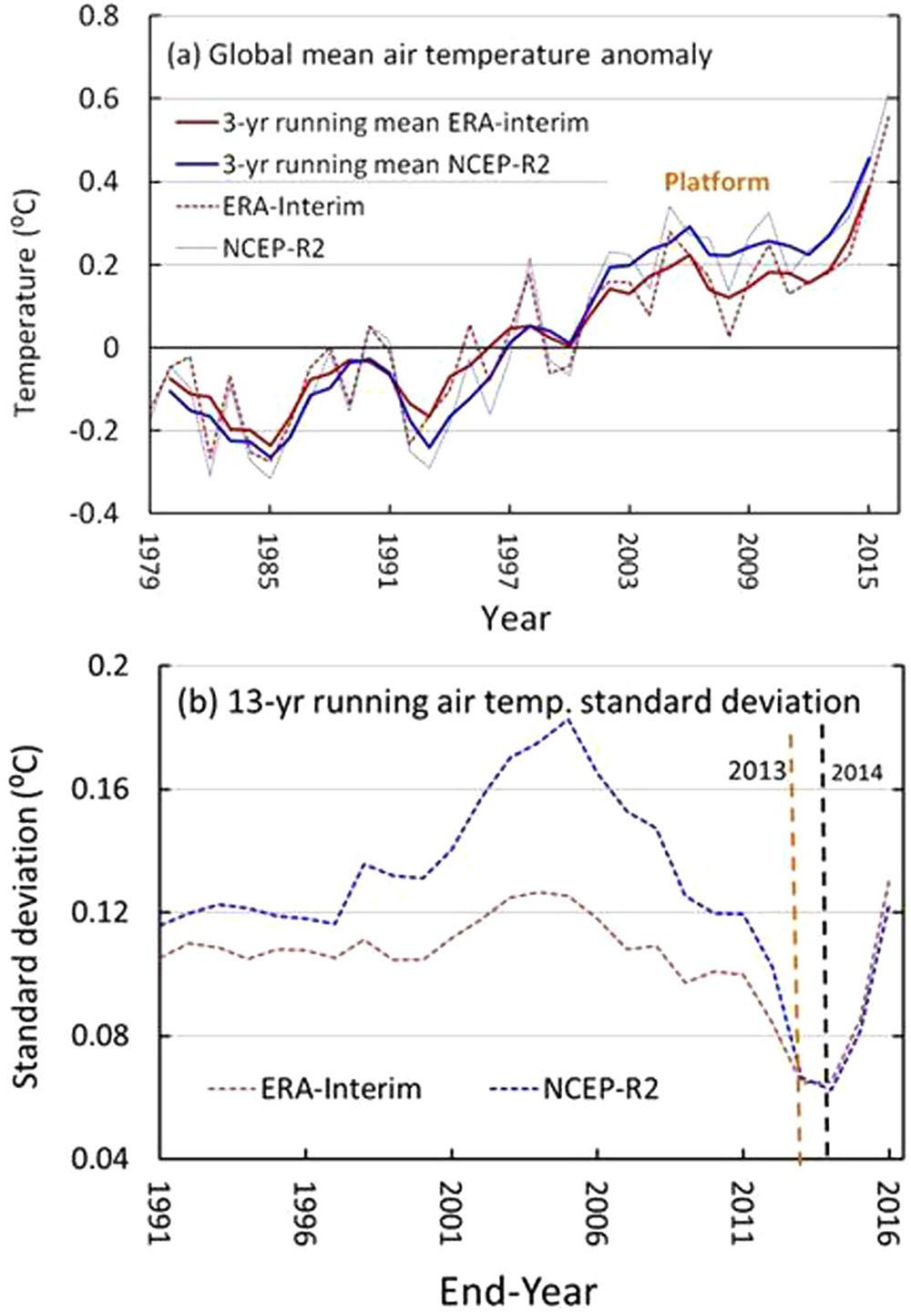
Global mean air temperature (GMAT) anomalies (for reference over the period 1981–2010) and their decadal standard deviations (STDEVs). (**a**) GMATs with the 3-yr running means of the ERA-Interim and NCEP-R2 reanalysis and (**b**) 13-yr running GMAT STDEVs. The figures were generated using Excel (https://www.microsoft.com/zh-cn/download/office.aspx).

In addition, a similar hiatus can also be found in the GMAT from the ERA-Interim and NCEP-R2 reanalyses, which are dynamically consistent and have full data coverage of the surface. Figure 4a shows that there is also a decadal platform similar to that observed for the GMST (Fig. 1a) during the first decade of the 21^st^ century, and a corresponding minimum STDEV appears for the period 2001–2013 for the ERA-Interim and for the period 2002–2014 for the NCEP-R2 in comparison with the trends over the larger or smaller window widths surrounding these periods (Fig. 4b). These results indicate that the interannual variability of the GMAT also became much weaker during the platform period than during previous decades when the interannual variability of the GMAT greatly intensified in approximately 2000. Hence, 2001–2014 can be regarded as a potential hiatus period to be tested further.

**Figure 5:**
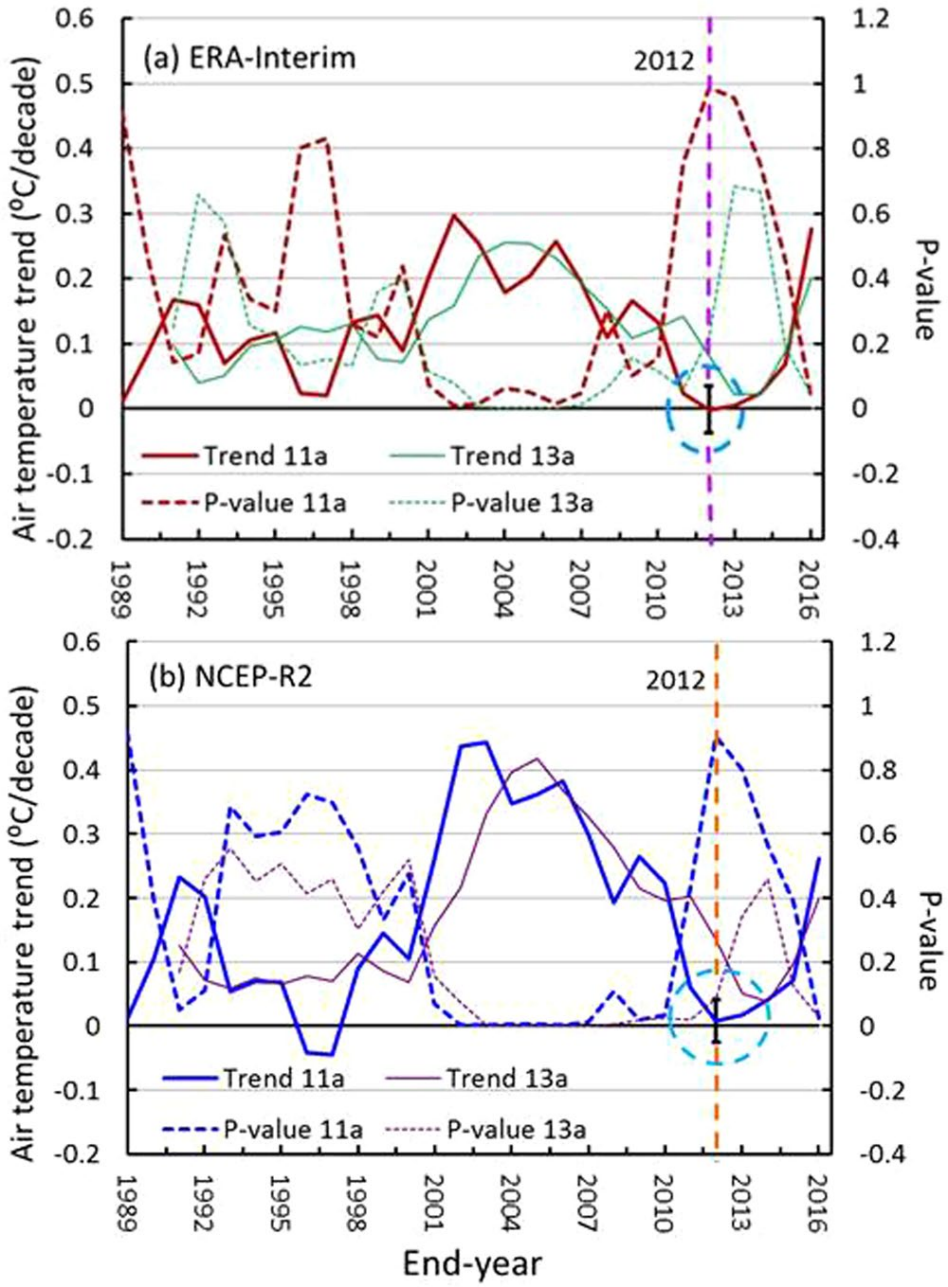
The 11-yr and 13-yr running trends and their P-values for global mean air temperature (GMAT) anomalies for (**a**) ERA-Interim and (**b**) NCEP-R2. The blue dashed circles represent the hiatus location, and the black bars represent their uncertainties (units: °C/decade). The figures were generated using Excel (https://www.microsoft.com/zh-cn/download/office.aspx).

Figure 5 shows that the 11- and 13-yr running trends in the GMAT series are similar to those of the GMSTs (Fig. 2a,b). The trends increased following the 1990s, reached their maximums in approximately 2000, and transitioned to decreasing to minimums over the period 2002–2012, and the corresponding P-values obtained via an F-test reached their maximums during the same period (2002–2012), which also contained their minimum STDEVs. The minimum trends for the two reanalysis datasets are also the smallest relative to those over wider windows, for example 12- or 13-yr windows. The minimum trend (with uncertainty) of the ERA-Interim dataset over the period 2002–2012 is approximately −0.0001 ± 0.0361 °C/decade, with a maximum P-value of 0.9885 (Table 1; Fig. 5a), while that of the NCEP-R2 dataset is 0.0082 ± 0.0337 °C/decade, with a maximum P-value of 0.9057 (Fig. 5b), which indicates that the minimum trends were the most nonsignificant. Over the period 2001–2013, the minimum trend is approximately 0.0216 ± 0.0259 °C/decade, with a maximum P-value of 0.6848 for the ERA-Interim dataset (Table 1; Fig. 5a), while the trend for the NCEP-R2 is 0.0377 ± 0.0245 °C/decade from 2002–2014 dataset, with a maximum P-value of 0.4581 (Fig. 5b). These trends are all greater than those from the period 2002–2012, and their corresponding P-values are also less than those from the period 2002–2012. Hence, the trends over the period 2002–2012 should be regarded as the early 2000s hiatuses based on the two reanalysis datasets because their minimum trends are all below 0.01 °C/decade.

### Multiscale decomposition

The hiatus is associated with contributions from temperature components at various scales, which can easily be associated with either external forces or the internal variability of the climate system. To include the reanalysis datasets, two new time series of the GMST from the period 1889/1901–2016 are established in addition to the five time series from the gridded HadCRUT4, Cowtan & Way, NOAA-old, NOAA-new and GISTEMP datasets. The first is a combination of the 20th-century Coupled European Centre for Medium-Range Weather (ECMWF) Reanalysis (CERA-20C) dataset and the ERA-Interim (hereafter CERA-Interim), and the second is created by merging the NCEP-R2 and NOAA-CIRES Twentieth Century Reanalysis (V2c) (NOAA20C-NCEP-R2, hereafter) after bias corrections between them (see data and methods and Supplementary materials), where the ERA-Interim or NCEP-R2 is regarded as the standard reference in the correction. The seven series are decomposed into a series of orthogonal wavelet components at the cascade scales of 2*a*, 4*a*, 8*a*, 16*a*, 32*a*, 64*a* (*a* refers to year in wavelet analysis) and beyond (i.e., nonlinear trends at the century scale) based on the Daubechies-4 (Daub4) wavelet basis^30^ (see data and methods). The components are sorted into three parts: the interannual composite, with the scale of 2–8a; the multidecadal composite, with the scale of 16–64a; and the nonlinear trend at the century scale. Here, the nonlinear trend may be defined as a component of global warming because it represents the evolution of temperature at the century scale since 1889. This is after 1870, which is regarded as the beginning of the global Industrial Revolution epoch.

### Global warming

**Figure 6:**
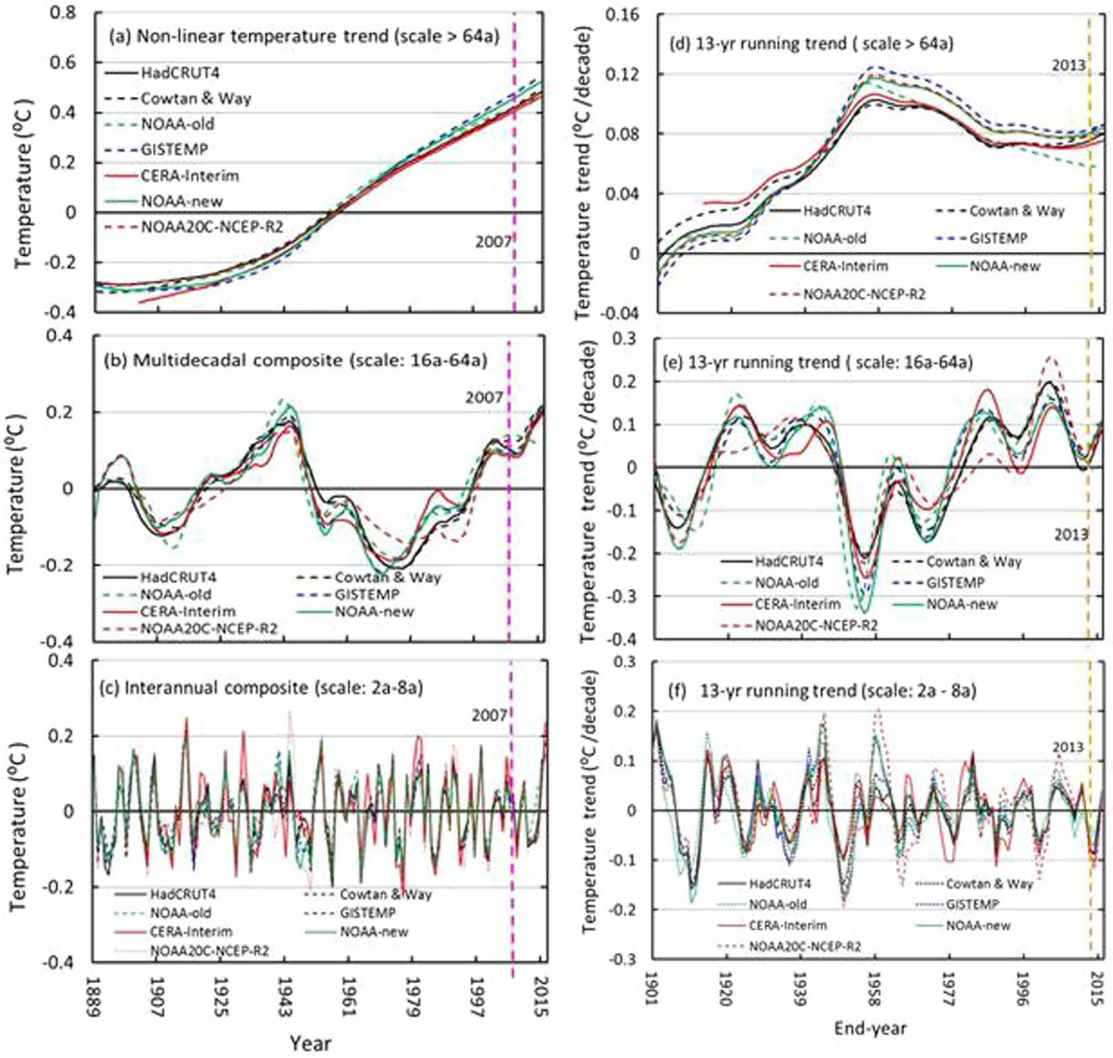
Orthogonal wavelet decomposition of the global mean surface temperature (GMST) and the 13-yr running trends during 1889–2016/1901–2016 via the HadCRUT4, Cowtan & Way, NOAA-old, NOAA-new, GISTEMP, CERA-Interim and NOAA20C-NCEP-R2 datasets. CERA-Interim are combined via the 1901–1978 CERA-20C and the 1979–2016 ERA-Interim datasets, and NOAA20C-NCEP-R2 is combined with 1889–1978 NOAA-CIRES Twentieth Century Reanalysis (V2c) and 1979–2016 NCEP-R2 (see data and methods). (**a**) Nonlinear trends with scales greater than 64a; (**b**) multidecadal composite (16–64a); (**c**) interannual composite (2–8a); and (**d**–**f**) 13-yr running trends of the three composites. The brown dashed line denotes the location of the end year (2013) of the hiatus, and the pink dashed line denotes the middle year (2007) of the hiatus for the period 2002–2012 or for the period 2001–2013. The figures were generated using Excel (https://www.microsoft.com/zh-cn/download/office.aspx).

Figure 6a shows that nonlinear trends increased monotonically from the 1900s to the 2000s, which implies that the global climate at the century scale warmed gradually over the past hundred years. Fig. 6d shows that global warming started in the 1900s rather than in 1870, which was the first year of the Industrial Revolution epoch, as is well known, and that the warming gradually accelerated towards its maximum value just after World War II (1950s). And the warming turned to declining until the 1980s and then exhibited almost constant trends for approximately three decades (excluding the trends in NOAA-old). Almost all of the maximum trends appeared over the period 1945–1957 in the 13-yr running trend, except for those in the NOAA-old, where a maximum trend was found for 1943–1955 (0.1142 °C/decade). Other maximum trends were 0.10267, 0.0998, 0.1175, 0.1250, 0.1065 and 0.1193 °C/decade for the HadCRUT4, Cowtan & Way, NOAA-new, GISTEMP, CERA-Interim and NOAA20C-NCEP-R2 datasets, respectively. In addition, their constant trends were approximately 0.08 °C/decade, on average, from the 1980s to the 2000s, while the NOAA-old trend kept decreasing during the last three decades at a rate similar to that during previous decades after World War II. There was no GWH in the early 2000s in the century-scale component of the GMSTs of the seven series. In addition, data infilling or bias correction in the SST measurements caused the NOAA-new trend to be higher than that of the NOAA-old in the component, while no significant difference was found among those from the 1940s–1990s for HadCRUT4 and Cowtan & Way, except for in the 2000s, when the latter’s trend became higher than the former’s, which may reflect accelerated warming in the Arctic region in recent decades. Furthermore, the trend of NOAA20C-NCEP-R2 is above that of CERA-Interim and coincides with the NOAA-new after World War II. The difference may result from the data assimilation systems of the two reanalysis datasets. As the relative strength of the warming differs before and after World War II, this may reflect the influences of the different interpolation approaches on the properties of the datasets, as there was extremely sparse data coverage in the early part of the time series.

### Interannual and multidecadal oscillations

Figure 6b shows that there is an apparent oscillation with a large magnitude at the scale of approximately 16–64 a for all the GMST series, in which the negative phases in the 1960s-1970s correspond to a relatively cool period in the global climate during the twentieth century, as is well known, and then the composites increased to a new maximums in 2016. As shown in Fig. 6a and b, the hot global climate experienced since the 1980s resulted from an overlap in global warming and a positive phase in the multidecadal composite, while the cool decades (1960s-1970s) appeared only with the negative phase of the multidecadal composite (Fig. 6b). However, Fig. 6b also shows that there are short-term oscillations in the composite, where the last oscillation coincides with the hiatus period (2001–2013/2002–2012) and the minimum trend from the period 2001–2013/2002–2012 for all of the series (Fig. 6e). This result indicates that the multidecadal composite makes a minimal contribution to the GMST trend during the hiatus period, except for that of the HadCRUT4, which exhibits a small cooling trend during the period that works against a trend of global warming. Hence, the global warming trend cannot be balanced by the trend in the multidecadal composite during this period. However, the variability of the interannual composite (2–8 a) becomes weak over the period 2001–2013, with an apparent cooling trend (Fig. 6c,f). By comparing Fig. 6d,e, and f, one can see that the early 2000s hiatus results from an overlap of the three trends (i.e., a nonlinear trend and those of the multidecadal and the interannual composites) over the period 2001–2013. Thus, the development of a hiatus or slowdown results primarily from the balance between the cooling of the interannual composite and global warming, which keeps the warming at a constant rate. Similar results can also be found from the time series for the period 2002–2012. Additionally, there is a significant difference between the multidecadal composites of the two reanalysis datasets and between their trends, which may reflect the different data assimilation systems and SSTs because the composite may be correlated with the interdecadal and multidecadal modes of climate, such as the Atlantic Multidecadal Oscillation (AMO)^35^, Pacific Decadal Oscillation (PDO)^36^ or Interdecadal Pacific Oscillation (IPO)^37^. Similarly, the differences between NOAA-new and NOAA-old are larger than those between HadCRUT4 and Cowtan & Way, reflecting the importance of bias correction in the SST measurement.

**Figure 7:**
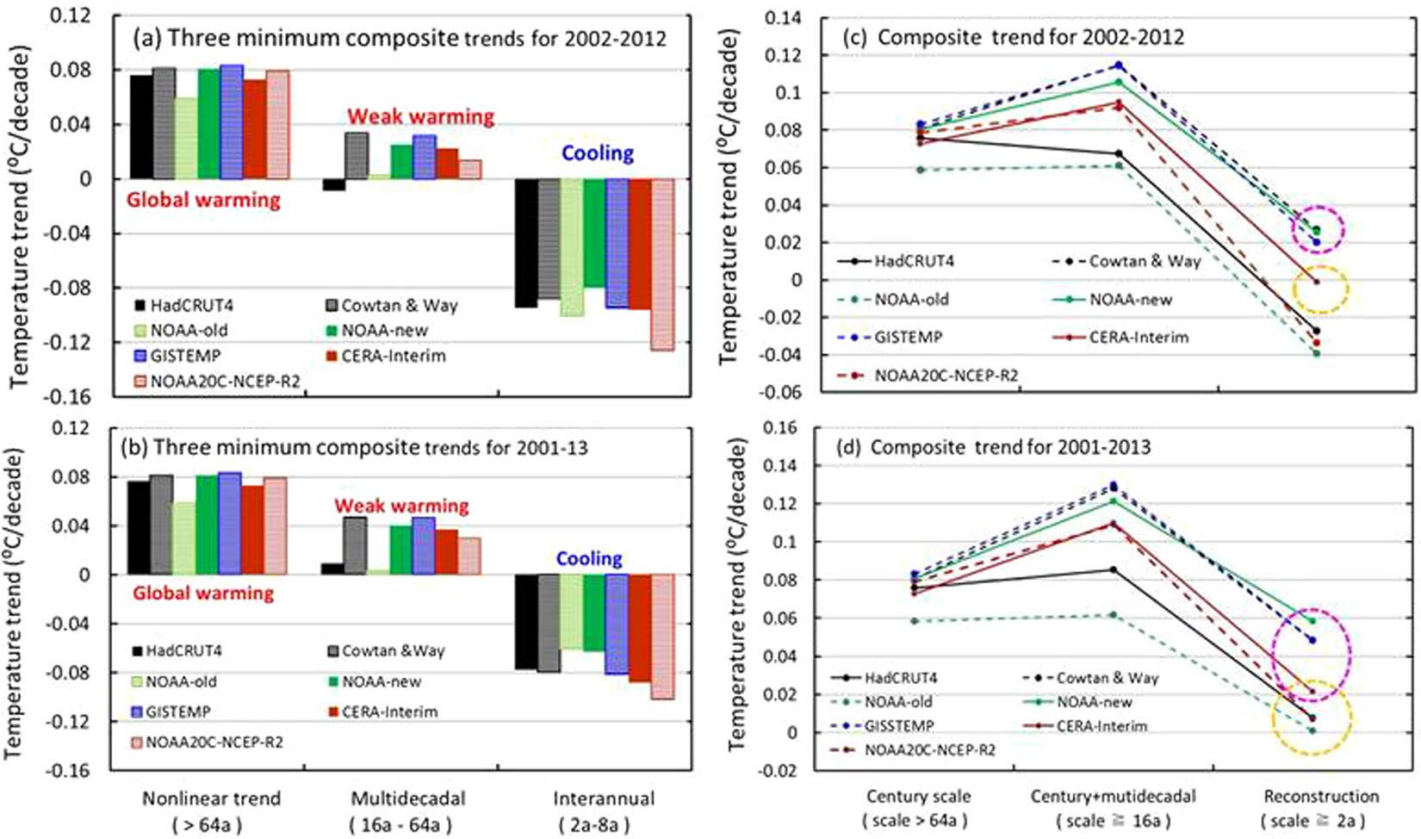
Minimum trends in the wavelet components of the seven GMST series during the early 2000s. (**a**) The component trends are for the period 2002–2012; (**b**) the component trends from 2001–2013; and (**c**) trends in the composites are for the period 2002–2012 and (**d**) trends in the composites are for the period 2001–2013. The points (i.e., trends) circled in Figures c and d with dashed yellow rings are regarded as the hiatuses, while those circled with pink dashed rings are regarded as the slowdowns (unit: °C/decade) in this study. The figures were generated using Excel (https://www.microsoft.com/zh-cn/download/office.aspx).

Figure 7a and b clearly show the differences in the three composite trends for the seven time series, which seemingly result from different interpolation approaches/data assimilation systems, bias corrections or numbers of observation records used. For example, the infilling and bias correction both increased warming and reduced cooling of the composite at the interannual scale from 2002–2012, but for 2001–2013. The increase in the trend in NOAA-new relative to NOAA-old is larger than that in Cowtan & Way relative to HadCRUT4, which reflects the effect of bias correction on the reported warming (Fig. 7). In addition, the infilling or bias correction has more influence on the trends in the multidecadal composites than in other composites over the same periods, implying that temperature changes in the oceans, Arctic region and African continent are important contributors to global mean climate change, which leads to the slowdown observed over the period 2002–2012 in the NOAA-new and Cowtan & Way (Fig. 7c) datasets rather than the hiatus observed over 2001–2013 in the HadCRUT4 and NOAA-old datasets (Fig. 7d). In addition, the hiatus can also be found in the CERA-Interim dataset over the period 2002–2012, which implies that infilling and bias correction may lead to overestimated warming trends in the interdecadal and multidecadal composites and underestimated cooling trends in the interannual composite during the early 2000s (Fig. 7a) if the reanalysis is taken as a reference, because the reanalysis is dynamically consistent and incorporates many more four-dimensional observation records (regular or irregular) using the state-of-the art four-dimensional data assimilation system^28^. This system is generally regarded as more advanced than any three-dimensional interpolation method used in gridded datasets. Thus, an early 2000s hiatus exists in the GMSTs of the HadCRUT4, NOAA-old and the reanalysis datasets during the periods 2002–2012/2001–2013, while the slowdown is found with slightly higher trends in the Cowtan & Way, NOAA-new and GISTEMP datasets.

**Figure 8:**
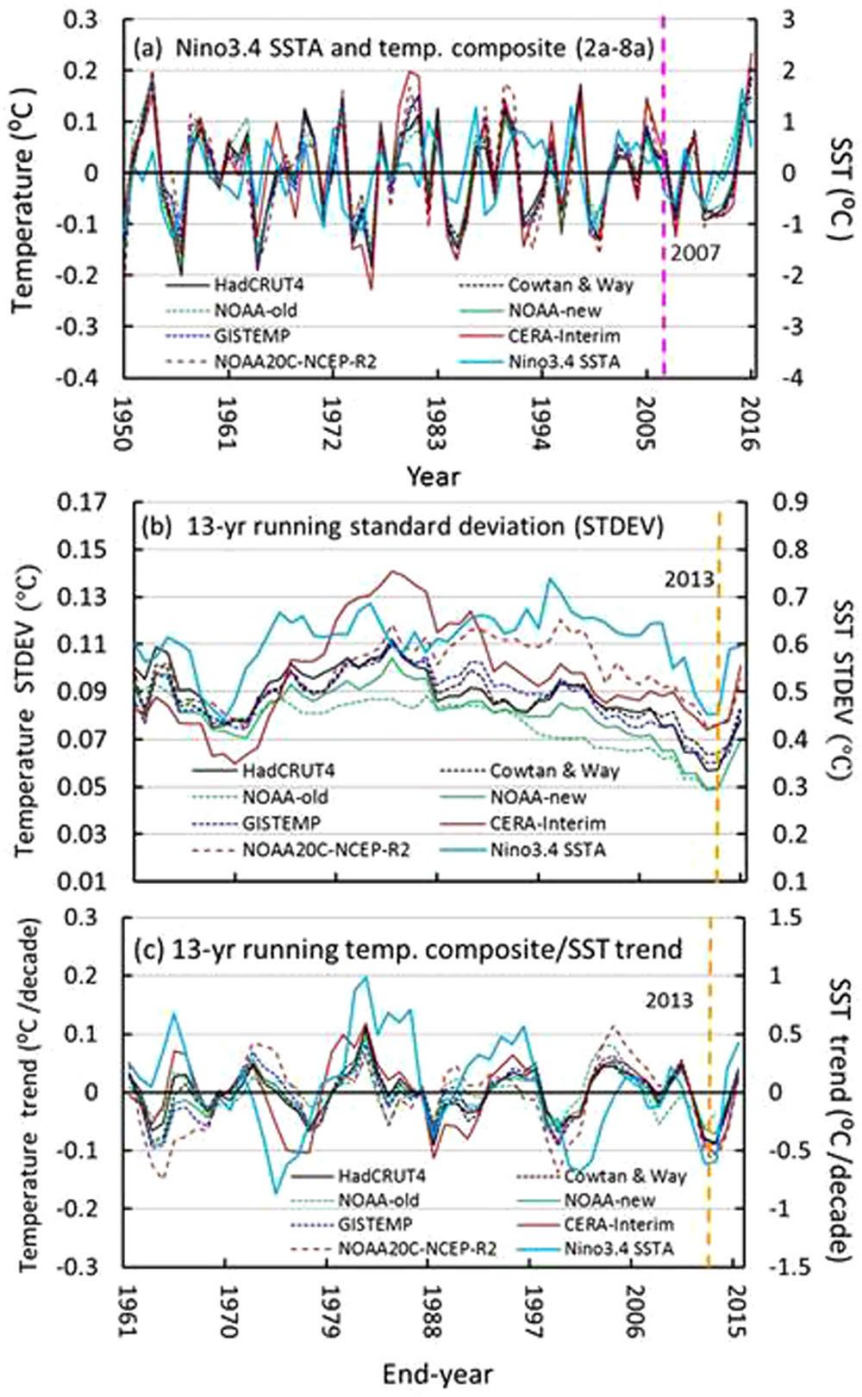
Interannual composites (2–8a) with 13-yr running standard deviations (STDEVs) and trends. (**a**) Interannual composites of the GMST series, with annual mean Niño3.4 SST anomalies; and (**b**,**c**) 13-yr running STDEVs and trends. The vertical pink dashed line indicates the middle year (2007) of the hiatus or the slowdown, and the vertical yellow dashed line denotes the end year (2013) of the STDEV or trend for the period 2001–2013. The figure was generated using Excel (https://www.microsoft.com/zh-cn/download/office.aspx).

Figure 8a shows that the evolutions of the interannual composites coincide well with the Niño3.4 SST anomalies for the period 1950–2016. Their correlation coefficients all exceed the critical value (0.317) at a significance level of α = 0.01 for an effective number of degrees of freedom (63; see data and methods). The coefficients are 0.480, 0.408, 0.404, 0.466, 0.381, 0.403 and 0.328 for the HadCRUT4, Cowtan & Way, NOAA-old, NOAA-new, GISTEMP, CERA-Interim and NOAA20C-NCEP-R2 datasets, respectively. The interannual variability of the GMST essentially results from an ENSO cycle that is typically described by Niño3.4 SSTA or the Niño 3 SST anomaly (see data and methods). There is also an extremely low STDEV for the period 2000–2013 for every temperature series and Niño3.4 SSTA during the second half of the twentieth century, except for CERA-Interim, in which extremely low STDEVs appeared approximately in the early 2000s and 1960s (Fig. 8b). This result reveals that the interannual variability of the GMST became extremely weak during the hiatus period (2001–2013), which was coupled with an extremely weak ENSO cycle in the east equatorial Pacific. Furthermore, the 13-yr running trends of the composites of the time series also coincide with the trends of the Niño3.4 SSTA^38,39^, especially those in the period 2001–2013. Hence, the cooling trend of the interannual composite most likely results from the ENSO cycle, because this result is consistent with the numerical experiment forced only by SSTAs in the east equatorial Pacific^5^. Calculation confirmed that the extreme cooling during the hiatus period results from warmer SST in the first half of the hiatus period (2001–2013) and cooler SST in the second half, which is associated with asymmetrical ENSO events around the middle of the period.

## Discussion

The early-2000s hiatus ended as soon as a sharply warming appeared in 2015–2016 with a new extreme El Niño event developing in the east equatorial Pacific. Under such circumstances, we quantitatively examined the existence of the hiatus and its duration in various data sources, including gridded and reanalysis datasets. The results demonstrate that the hiatus has a decadal duration with minimum STDEV on average and a near-zero trend over 2002–2012/2001–2013/2002–2014 at the most nonsignificant level that corresponds to the maximum P-value obtained via an F-test. The hiatus identified here differs from that shown in published literature^3–6^, in which the hiatus or slowdown began with the great 1997/1998 El Niño event^40^, as is well known. The hiatus of 2002–2012/2002–2014 is found in the GMST/GMAT of ERA-interim and NCEP-R2 datasets, while that in the period 2001–2013 is in the GMSTs of HadCRUT4, NOAA-old. The minimum trends for the period 2002–2012 in the NOAA-new, GISTEMP and Cowtan & Way datasets are slightly higher than their counterparts in the NOAA-old and HadCRUT4 datasets, as a result of the infilling of data coverage and the bias correction of the SSTs, and their trends over the period 2002–2012 are thus regarded as slowdowns, following prior arguments on the definition of the hiatus^17,21^. A slowdown may be also regarded as a hiatus if the trends (0.0255, 0.02 and 0.0482 °C/decade) are considered smaller or near zero. As the 2002–2012 hiatus is included in the 2001–2013 or 2002–2014 or 2002–2012 periods, the last can be suggested as the common hiatus duration in the all GMSTs/GMATs of the nine time series included in this study. Additionally, statistical analysis reveals that the hiatus or slowdown was accompanied by a minimum STDEV, which is an additional characteristic of the hiatus that indicates that the near-zero trend over the hiatus period resulted from a platform-like segment of the time series rather than a decadal valley or ridge of a GMST/GMAT wave.

Multiscale decomposition reveals that the hiatus essentially results from a decadal balance between cooling from the interannual composite and global warming, in addition to weak warming from the interdecadal and multidecadal composite because their maximum magnitudes appeared in the positive phase after 2000. This is somewhat different from the argument that proposes the negative phase of the Interdecadal Pacific Oscillation (IPO) as the major mechanism for hiatus formation on PCA analysis^6^. Further decomposition shows that only the interdecadal component (scale: 16 a) makes a small contribution (through cooling) to the hiatus, while the multidecadal composite contributes weak warming. The most important finding is that the variability of the interannual composite well coincides with the Niño3.4 SST anomaly, which is of almost the same statistical characteristics as the composites, such as the running STDEV and trend, especially over the period 2001–2013 (Fig. 8b,c). This indicates that the interannual variability of the GMST is coupled with the ENSO cycle^38,39^, and thus, the hiatus results mainly from the east equatorial Pacific SST anomalies^41^, as the numerical experiment that reproduced the early 2000s hiatus was performed by using a climate model forced only by the SST anomalies in the east equatorial Pacific^5^.

Figure 8b and c also shows that there were several cooling events at decadal or even multidecadal scales during the period 1950–2016, while there were three minimum trends in the interdecadal and multidecadal composite (Fig. 6e), in which only the last one encountered strong cooling from the interannual composite during the 2000s, which implies that the early-2000s hiatus was a transient event, in comparison with the long-term cooling event observed during 1960s-1970s, which was caused by the negative phase of the interdecadal and multidecadal composite (Fig. 6b) with extreme cooling that was much stronger than global warming at a two-decade scale during this period (Fig. 6b,e). Figure 6b also shows that the multidecadal composites reached their maximums in magnitude in 2016 and then will probably turn onto a downward path, i.e., a cooling phase will soon be observed. This suggests the beginning of a new multidecadal cycle of global climate similar to that experienced in the second half of the twentieth century following the hiatus. Hence, future multidecadal climate changes should depend on the competition between global warming and cooling at the multidecadal scale if global climate repeats the last cycle observed at multidecadal scales in the coming decades.

### Data and methods

Seven GMST series and two GMAT anomalies were used in this investigation, including the 1889–2016 HadCRUT4 dataset^18,19^, which was downloaded on 10/10/2017 from the Climate Research Unit (CRU) at the University of East Anglia (UEA, https://crudata.uea.ac.uk/~timo/diag/tempdiag.htm), along with its updated dataset (version 2) that includes infilling over the Arctic region and African continent (hereafter, Cowtan & Way^17^), which was downloaded on 18/11/2017 from the Department of Chemistry at the University of York (http://www-users.york.ac.uk/~kdc3/papers/coverage2013/had4_krig_annual_v2_0_0.txt); the old version^21^ of the bias-corrected GMST series from 1887 to 2014 (hereafter NOAA-old), or NOAA’s merged land-ocean surface temperature dataset, which was downloaded on 16/07/2015 at ftp://ftp.ncdc.noaa.gov/pub/data/scpub201506/OldAnalysis/, and its new version, which is the bias-corrected GMST series (hereafter, NOAA-new^23^) that was downloaded on 17/11/2017 from the NOAA National Centers for Environmental Information (NCEI) (https://www1.ncdc.noaa.gov/pub/data/noaaglobaltemp/operational/timeseries/); the GISS Surface Temperature Analysis (GISTEMP Team, 2018) dataset from the NASA Goddard Institute for Space Studies, where the dataset was accessed on 18/11/2017 at https://data.giss.nasa.gov/gistemp/; and the 1979–2016 GMST/GMAT time series via the ERA-Interim dataset (hereafter, ERA-Interim), which combined land temperatures at 2 m and SSTs at 1°x1° meridional and latitudinal grids^28^ and was downloaded on 06/11/2017 from the ECMWF at http://apps.ecmwf.int/datasets/data/interim-full-moda/levtype=sfc/. In addition, GMST/GMAT anomaly series are calculated from the reanalysis dataset of NCEP-DOE Reanalysis 2 (hereafter NCEP-R2)^29^ with a resolution of 192 × 194 on a Gaussian grid, which was provided by the NOAA/Oceanic and Atmosphere Research/Earth System Research Laboratory (NOAA/OAR/ESRL) Physical Sciences Division (PSD) in Boulder, Colorado, USA and downloaded on 28/11/2017 from http://www.esrl.noaa.gov/psd/. To compare the multiscale characteristics of the gridded datasets with those of the reanalysis, a twenty-first century reanalysis dataset (CERA-20C, 1901–2010) was introduced with 1° × 1° meridional and latitudinal grids, which was downloaded on 04/02/2017 from ECMWF at http://apps.ecmwf.int/datasets/data/cera20c/levtype=sfc/type=an/. Thus, a new reanalysis series from 1901–2016 was established by combining the GMSTs of the CERA-20C and ERA-Interim datasets, where the 1979–2016 period was infilled with ERA-Interim GMST and the 1901–1978 period was infilled with CERA-20C (hereafter CERA-Interim) with bias correction of the mean difference between their GMSTs for the period 1979–1983, because their difference gradually decreased over time from 1979 to 2010. A similar strategy was employed in establishing the NOAA20C-NCEP-R2, which is a combination of the 1979–2016 NCEP-R2 and 1889–1978 NOAA-CIRES Twentieth Century Reanalysis Version 2c, using resources of the National Energy Research Scientific Computing Center managed by Lawrence Berkeley National Laboratory and supported by the Office of Science of the U.S. Department of Energy under Contract No. DE-AC02–05CH11231 (Support for the Twentieth Century Reanalysis Project version 2c dataset is provided by the U.S. Department of Energy, Office of Science Biological and Environmental Research (BER), and by the National Oceanic and Atmospheric Administration Climate Program Office)^42–47^, and the dataset was downloaded from https://www.esrl.noaa.gov/psd/data/gridded/data.20thC_ReanV2c.monolevel.mm.html on 21/07/2018. In addition, the Niño3.4 SSTA is represented by the averaged SSTA over the east equatorial Pacific (5°N-5°S, 170°W-120°W), which was obtained from the Global Climate Observing System (GCOS) Working Group on Surface Pressure (WG-SP) at https://www.esrl.noaa.gov/psd/gcos_wgsp/Timeseries/Nino34/ on 20/01/2018.

The linear trend in temperature within a moving window is estimated using a linear regression (Excel function: *slope*) based on the *OLS* method^48–50^, which is used to search for the location of the hiatus or slowdown. The null hypothesis is that no trend exists, and the significance of the trend is measured by P-values obtained via an F-test using an effective degree of freedom *Ne*. *Ne* is estimated as follows

1 $${N}_{e}=N(\frac{1-\rho }{1+\rho }),$$

where *N* represents the length of the series, and ρ is the first-order autocorrelation coefficient. The uncertainty of the trend estimate can be approximated by the following formula^21^

2 $${S}_{tr}={[\frac{{S}_{e}^{2}}{{\sum }_{t=1}^{N}{(t-\bar{t})}^{2}}]}^{1/2},$$

where $\bar{t}$ represents the arithmetic mean of *t*, and ${S}_{e}^{2}$ represents the error variance, which is defined as

3 $${S}_{e}^{2}=\frac{1}{N-2}{\sum }_{t=1}^{N}{e}^{2}(t),$$

where *e*^2^(t) represents the residual of the linear regression equation, and *N* represents the sample size rather than the corresponding effective degree of freedom, *N*_*e*_^21^, due to the small sample size in the moving window in which the trend is estimated. The decadal STDEV is calculated with the Excel function *STDEV*.

In addition, the seven long-term GMST time series involved are also decomposed into series of orthogonal wavelet components at cascading scales of 2a, 4a, 8a, 16a, 32a, 64a and beyond (i.e., the century scale) for 128 sampling points (1889–2016/1887–2014) based on the orthogonal wavelet decomposition with a regional basis of Daub4^30^.

The signal *S*(*t*) can be reconstructed as

4 $${\rm{S}}({\rm{t}})={A}_{5}+{\sum }_{k=1}^{5}{D}_{k},$$

where *D*_*k*_ represents the k-th detail of the signal at decomposition level k, and A_5_ represents the approximate signal at the highest level (5) for 128 samples, which is usually regarded as the nonlinear trend in the signal. The wavelet time scales of (2–8a), (16–64a) and beyond 64a represent the interannual scales, multidecadal scales and the scales beyond 64a, respectively. Here, the last one (A_5_) represents the global warming component of the GMST for 128 samples (1889–2016 or 1887–2014 for the NOAA-old dataset). The scale is usually proportional to the period of a periodic signal. The wavelet decomposition is conducted using Python^45^ (https://www.python.org/).

## Electronic supplementary material

Data 1a

Data 1b

Data 2a

Data 2b

Data 2c

Data 2d

Data 3a

Data 3b

Data 3c

Data 4a

Data 4b

Data 5a

Data 5b

Data 6a

Data 6b

Data 6c

Data 6d

Data 6e

Data 6f

Data 7a

Data 7b

Data 7c

Data 7d

Data 8a

Data 8b

Data 8c
